# White Spots Prevalence and Tooth Brush Habits during Orthodontic Treatment

**DOI:** 10.3390/healthcare10020320

**Published:** 2022-02-08

**Authors:** Çeljana Toti, Agron Meto, Gerta Kaçani, Etleva Droboniku, Dorjan Hysi, Michele Tepedino, Edlira Zaja, Luca Fiorillo, Aida Meto, Denada Buci, Olja Tanellari

**Affiliations:** 1 Department of Orthodontics, Faculty of Dental Medicine, University of Medicine, 1005 Tirana, Albania; gerta.kacani@umed.edu.al (G.K.); oli_koca@yahoo.com (O.T.); 2 Department of Dentistry, University of Aldent, 1000 Tirana, Albania; agronmeto@yahoo.com (A.M.); lfiorillo@unime.it (L.F.); 3 Department of Dental Therapy, Faculty of Dental Medicine, University of Medicine, 1005 Tirana, Albania; etleva.droboniku@umed.edu.al (E.D.); dorjan.hysi@umed.edu.al (D.H.); 4 Department of Biotechnological and Applied Clinical Sciences, University of L’Aquila, 67100 L’Aquila, Italy; m.tepedino@hotmail.it; 5 Department of Public Health, University of Medicine, 1005 Tirana, Albania; edlirazaja@gmail.com; 6 Department of Biomedical and Dental Sciences, Morphological and Functional Images, University of Messina, 98100 Messina, Italy; 7 Multidisciplinary Department of Medical-Surgical and Odontostomatological Specialties, University of Campania “Luigi Vanvitelli”, 80121 Naples, Italy; 8 Endodontic Clinical Section, School of Dentistry, Department of Biomedical and Neuromotor Sciences, University of Bologna, 40125 Bologna, Italy; 9 Independent Researcher, 1000 Tirana, Albania; denadabuci@hotmail.com

**Keywords:** white spots, oral health, orthodontics, enamel tooth demineralization

## Abstract

White spots (WS) are one of the most undesirable side effects in patients undergoing orthodontic therapy and are usually located around bracket bases and even detected under the molar bands. The aim of the present cross-sectional study was to evaluate the WS lesion during orthodontic therapy and the correlation between WS and oral hygiene habits. Patients requiring orthodontic treatment with a fixed appliance were screened for the inclusion/exclusion criteria, and 74 subjects were finally enrolled. Each patient received three examinations: at T0, the day of the application of the fixed appliance; at T1, three months later; and at T2, six months after treatment start. After calculating descriptive statistics, differences between groups were evaluated with an independent sample *t*-test. The first type error was set as *p* ≤ 0.01. The observed prevalence of WS lesions was 59.5% on T1 and 60.8% on T2. The most affected teeth result to be upper molars, lower left first molar, upper right central incisor and upper left lateral incisor, upper right canine, upper left first premolar, and lower right first molar. A higher frequency of daily tooth brushing was accompanied by a lower prevalence of WS. No significant effect of sex was observed.

## 1. Introduction

White spot (WS) lesions are areas of local decalcification of enamel without cavity formation and are defined as “subsurface enamel porosity from carious demineralization” that is presented as “a milky white opacity” when located on smooth surfaces [1,2].

WS are one of the most undesirable side effects in patients undergoing orthodontic therapy. Several studies have demonstrated that WS become visible during the first weeks of orthodontic treatment [3,4]. They are usually located around bracket bases, half-moon shaped, but they can also be detected under the molar bands and sometimes can even look like linear defects along the marginal surface of a band [5,6]. Brackets, bands, wires, and attachments used during fixed orthodontic therapy, due to their complex design, limits plaque removal and self cleaning. They contribute to the adhesion of oral plaque leading to enamel demineralization [7].

WS are described as white opaque areas caused by enamel demineralization and are usually located underneath the dental plaque [8,9]. This is attributed to the change in optical properties of light reflection of the decalcified enamel [8]. WS might remineralize over time, but the enamel will not recover its original aspect, thus leading to an unpleasant appearance of the teeth [10,11]. The increase of enamel porosity will facilitate bacteria penetration, thus preventing remineralization [12]. Calcium and phosphate ions that are in this surface can hardly penetrate in between interprismatic spaces to help with the remineralization process [10]. Similarly, salivary proteins that assist in inhibiting the demineralization process (proteins rich in proline and statherine) cannot easily penetrate in between these pores formed in the enamel. As a result, this sub-superficial demineralization changes the refractory index of the enamel and is clinically manifested as a milky-white opacity area otherwise known as WS [13,14]. According to Gorelick, L.; et al. [5] who studied the incidence of WS lesion in orthodontic patients, the 50% of patients develop at least one WS lesion during their orthodontic treatment. Other studies [6,14,15] demonstrated how the prevalence and the severity (opacity index) of WS would increase in proportion with the prolonging in time of the orthodontic treatment.

There is variation from 2% to 96% in the prevalence of WS, depending on the technique of examination [5,10,14]. WS might be carious or non-carious lesions. WS as carious lesions are clinically presented as rough, opaque, and porous lesions, while WS as non-carious lesions are smooth and polished looking [8,16]. Non carious WS lesion include fluorosis, enamel hypomiralization, and enamel hypoplasia. The incidence of WS and caries is significantly influenced by dietary, environmental, and socioeconomic factors, as well as oral hygiene habits [15,16]. Therefore, different populations may show even large differences in incidence and prevalence. There is one study conducted in Albania about molar-incisor hypomineralisation (MIH) among children aged 8–10 years [17], but there is no evidence regarding the occurrence of WS during orthodontic treatment. WS are present during orthodontic treatment, but we do not have evidence if there is a difference in the prevalence of these lesions before and during treatment.

Therefore, the aim of the study was to evaluate the WS lesion prevalence during orthodontic therapy. The correlation between WS and tooth brush habits was also investigated.

## 2. Materials and Methods

### 2.1. Patients’ Selection

This study was conducted during January to December 2019. The protocol of the study was approved by the Local Ethical Committee (Protocol no. 361/2019) and followed the recommendations provided by the Declaration of Helsinki from 1975 and subsequent revisions. All of the patients provided their consent after being informed at the beginning of the procedures. For underage patients, consent was obtained from their parents.

Patients requiring an orthodontic treatment with a fixed appliance were examined in a Private Dental Practice and screened for the following inclusion criteria: age above 11 years old, full permanent dentition, and orthodontic treatment with metallic fixed appliance placed on the vestibular surface of the teeth. In addition, the exclusion criteria were: patients that refused to sign the informed consent, patients with lip cleft lip and palate; patients with oro-facial syndromes; patients with congenital enamel defects (amelogenesis imperfecta, MIH, as defined by the European Academy of Pediatric Dentistry guidelines) [18]; and patients with restorations on the vestibular surface of the teeth. Sample size calculation for an independent samples *t*-test revealed that 58 subjects were needed to detect a large effect size of 0.8 with a power of 95% and a type I error of 1%. Seventy-four eligible patients were selected for the study and they were interviewed on their personal socio-demographic data including full name, age, gender, and their tooth brushing habits.

Each patient received three examinations: at T0, the day of the application of the fixed appliance; at T1, three months later; and at T2, six months after treatment start. Patients received oral hygiene instructions during each appointment.

### 2.2. The Examination of Teeth Surfaces

Examination for WS was performed following the International Caries Detection and Assessment System (ICDAS) protocols by two professionals that were trained and calibrated for the procedure. Calibration was performed over 27 patients (not included in the present investigation) to evaluate inter-examiner agreement through Kappa statistics, which revealed an almost perfect agreement (Kappa = 0.88). At T0, teeth were first cleaned with prophylactic paste and then isolated with cotton rolls, air-dried for 5 s, and examined under dental chair light. The examination of vestibular surfaces of maxillary and mandibular teeth was performed by starting from first molar on the right, ending on the first molar on the left. During the WS examination at T1 and T2 the bands were removed, the teeth were cleaned and examined. WS lesions were coded according to ICDAS assigning a score from 0 to 2 (Table 1). During each examination, routine clinical photographs were also taken. The data about WS were recorded in the clinical file of each patient.

### 2.3. Statistical Analysis

The data were analyzed by using the Statistical Package for Social Sciences (IBM SPSS Statistics for Windows, Version 26.0. Armonk, NY, USA: IBM Corp). Differences between groups were evaluated with an independent samples Students’ *t*-test. Statistical significance was set at *p* ≤ 0.01. The distribution of observed WS lesion at different ages according to gender, and for different tooth brushing habits was investigated through boxplots.

## 3. Results

### 3.1. Prevalence of WS Lesion

Among the 74 patients responding to the inclusion/exclusion criteria and finally enrolled in this study, 45 of them (60.8%) were females and 29 (39.2%) were males. The mean age of the sample was 17.4 ± 5.1 (range 11–33). The prevalence obtained of WS lesion was 59.5% in T1 and 60.8% in T2. *p* Value is 0.386, that means there is no significant difference between T1 and T2 on WS (Table 2).

Table 3 shows the distribution of WS according to gender at T1 and T2, respectively.

It was found that there were no significant differences between males and females regarding the presence of WS at T1 or T2.

### 3.2. Distribution of WS Lesion on the Affected Teeth

The distribution on the affected teeth is shown in Table 4.

Interestingly, the most affected teeth result to be UL first molar (15%), UR first molar (11%), LL first molar (10%), UR central incisor and UL lateral incisor (7%), UR canine, UL first premolar and LR first molar (6%). An inconsiderable number of WS lesions was observed on other teeth.

### 3.3. Correlation of Tooth Brushing Habits Related to WS Lesion

According to what patients declared, tooth brushing was performed from 0 to 4 times per day. Table 5 shows the tooth brushing frequency for 74 patients accordingly.

It was not found a statistically significant difference about tooth brushing frequency according to gender (Table 6).

**Figure 1:**
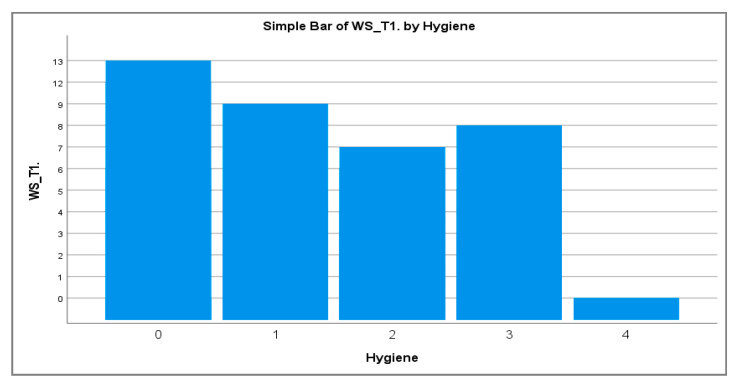
The association of tooth brushing frequency related with the WS number at T1.

The distribution of WS lesions among different oral hygiene regimen (frequency of daily tooth brushing) is shown in Figure 1. A higher frequency of daily tooth brushing was associated to a lower number of observed WS lesions at T1.

## 4. Discussion

In the present study, the clinical examination, under dental chair light revealed a high prevalence of WS formation during orthodontic treatment (59.5% at T1 and 60.8% at T2), and showed that a higher frequency of oral hygiene maneuvers had a protective effect on the development of WS lesions. This indicated that WS lesions are undoubtedly a major clinical problem in relation to treatment with fixed orthodontic appliances. The high prevalence of WS formation in this study indicated that these lesions are undoubtedly a major clinical problem in relation to treatment with fixed orthodontic appliances. Mizrahi was the one that found a significant rise of the WS prevalence from 72.3% to 84% in patients with fixed orthodontic appliances [14]. Boersma, J.G.; et al., registered a prevalence of 97% by using the light fluorescence examination technique [19].

Our study demonstrated also that there were no significant differences between males and females regarding the presence of WS. Females showed a higher presence of WS during orthodontic treatment compared to males, but this difference was not statistically significant. The study conducted by Mizrahi, E. [14] concluded that there was a significant difference between male and female. Males had an increase in lesion opacity index greater than females. However, there was no significant difference in the prevalence of WS, related to the gender of patients both before and after treatment with fixed orthodontic appliances. Similar gender-related data were found in other studies [15].

We found that WS lesions during orthodontic treatment were more common in the maxilla. In the maxilla, UR and UL first molars, UL central and lateral incisors, were the most affected teeth. Whereas in the mandible, the LL and LR first molar were the most affected teeth. Lower level of saliva over teeth in maxilla and bigger contact of bands might have influenced the difference of these lesion distribution.

Other authors noticed that these lesions are usually symmetrical and more common in the lateral incisors and maxillary canines as well as in the mandibular canines [10]. According to Gorelick, L.; et al. maxillary lateral incisors had the highest incidence to develop WS [5], while Geiger, A.M.; et al. stated that the maxillary lateral incisors and canines were the most affected teeth [20]. Other studies have shown that the most affected teeth were the first permanent molars, the maxillary incisors, the lateral incisors and the mandibular canines [21,22].

In our study, the data about tooth brushing habits was based on patients’ answers about the frequency of brushing. Most of patients (58.1%) under orthodontic treatment reported to brush their teeth twice a day. It was found that a higher frequency of daily tooth brushing was associated to a lower number of observed WS lesions at T1. Kühnisch, J.; et. al. [23] recommended that eliminating dental biofilm twice daily by tooth brushing with fluoride-containing toothpastes prevents the appearance of new carious lesions. An in vitro study, provided initial evidences that the combination of two innovative oral hygiene tools, may profoundly inhibit adhesion of oral cavity microorganisms onto orthodontic elastics. Also, their growth and biofilm formation onto their surfaces were greatly affected [24].

The observed prevalence of WS lesion in this study was 59.5% after three months of orthodontic treatment (T1) and increased to 60.8% six months after the orthodontic treatment (T2). Some studies found no association between treatment time and prevalence of WS lesions [25,26,27,28,29]. However, there are different attitudes and perceptions from different authors regarding the duration of treatment and the presence of WS during orthodontic therapy. Julien, K.C.; et al., [15] used a different observation period (24–36 months) for patients undergoing orthodontic therapy. They came to the conclusion that the duration of treatment affected the number of lesions on the teeth. According to a clinical study conducted [10], it was found a rapid increase in the number of WS appearing in the first 6 months of treatment and a slowdown in the rate of occurrence of these lesions for the next 12 months.

Finally, it is worth noting that the treatment of WS lesions still represents a challenge when striving for aesthetic perfection. However, the aesthetic improvement of both superficial and deeper lesions, is imperceptible. The evaluation of the patient’s oral hygiene before treatment start, as well as a customized prevention regimen for patients at higher risk, is of great importance [29,30,31,32,33,34].

One of the limitations of the present study was that WS were evaluated through visual examination under dental chair light, a technique less sensitive than the use of several optical techniques during recent decades, such as the optical caries monitor, use of quantitative laser and light- induced fluorescence, digital imaging with fiber-optic transillumination, laser fluorescence, and computer analysis of digital photographs. A bigger number of patients selected accorded to age may provide important information about WS correlated to age and tooth brush habits. However, it still represents a reliable first-line examination that is capable to provide a useful insight into the relevance of the risk of WS formation in orthodontic patients. In addition, since the T2 evaluation was performed when the orthodontic appliance was still in place, it is possible that the prevalence of WS might have been underestimated, since WS lesions located under the brackets and/or bands might not have been detected.

## 5. Conclusions

The prevalence of WS lesion was high three months after orthodontic treatment started and increased six months after the treatment started. The most frequently affected teeth were the UR and UL first molars, the LL first molar, the UR central incisor and the UL lateral incisor. A higher frequency of daily tooth brushing was accompanied by a lower prevalence of WS. No significant effect of sex was observed. The early diagnosis of WS is of critical importance considering how quickly these lesions can develop and become irreversible.

## Figures and Tables

**Table 1:** Visual examination criteria according to ICDAS.

Score	Examination Criteria
0	Sound tooth surface when probing: no evidence of caries after 5 s air drying
1	First visual change in enamel: opacity or discoloration (white or brown) is visible at the entrance to the pit or fissure after prolonged air drying
2	Distinct visual change is enamel visible when wet: lesion must still be visible when dry

**Table 2:** Prevalence of WS at T1 and T2.

WS	T1		T2		*p*-Value
	Frequency	Percent (%)	Frequency	Percent (%)	
0	30	40.5	29	39.2	*p* = 0.386
1	10	13.5	9	12.2	
2	9	12.2	10	13.5	
3	10	13.5	6	8.1	
4	3	4.1	6	8.1	
5	3	4.1	3	4.1	
6	3	4.1	2	2.7	
7	2	2.7	3	4.1	
8	1	1.4	1	1.4	
9	1	1.4	3	4.1	
10	0	0	1	1.4	
12	1	1.4	0	0	
13	1	1.4	1	1.4	
Total	74	100.0	74	100	

**Table 3:** The distribution of WS at T1 and T2 for males and females, respectively.

WS		0	1	2	3	4	5	6	7	8	9	10	12	13	Total	*p* Value
T1	M	13	5	3	3	0	1	2	0	1	0	0	0	0	29	*p* = 0.043
	F	17	5	6	7	3	2	1	2	0	1	0	1	1	45	
T2	M	11	6	4	2	1	1	2	0	0	1	0	0	1	29	*p* = 0.060
	F	18	3	6	4	5	2	0	3	1	2	1	0	0	45	

**Table 4:** The frequency of distribution of the affected teeth with WS lesion at T1.

upper right (UR) central incisor	11	7%
upper right (UR) lateral incisor	8	5%
upper right (UR) canine (cuspid)	9	6%
upper right (UR) first premolar (first bicuspid)	4	3%
upper right (UR) second premolar (second bicuspid)	2	1%
upper right (UR) first molar	**17**	**11%**
upper left (UL) central incisor	**13**	**8%**
upper left (UL) lateral incisor	**11**	**7%**
upper left (UL) canine (cuspid)	4	3%
upper left (UL) first premolar (first bicuspid)	9	6%
upper left (UL) second premolar (second bicuspid)	7	4%
upper left (UL) first molar	**23**	**15%**
lower left (UL) central incisor	2	1%
lower left (UL) first premolar (first bicuspid)	2	1%
lower left (UL) second premolar (second bicuspid)	1	1%
lower left (LL) first molar	**16**	**10%**
lower right (LR) central incisor	1	1%
lower right (LR) lateral incisor	1	1%
lower right (LR) canine (cuspid)	2	1%
lower right (LR) first premolar (first bicuspid)	2	1%
lower right (LR) second premolar (second bicuspid)	2	1%
lower right (LR) first molar	9	6%

Bold is used to distinguish them from the other teeth analyzed.

**Table 5:** Tooth brushing frequency for selected patients.

Tooth Brushing Frequency	Patients	Percent (%)
0	6	8.1
1	13	18.9
2	41	58.1
3	8	12.2
4	1	2.7
Total	74	100.0

**Table 6:** Tooth brushing frequency according to gender.

Gender	Tooth Brushing Frequency					Total	*p* Value
	0	1	2	3	4		
M	2	9	16	1	1	29	
F	4	5	27	8	1	45	*p* = 0.139
Total	6	14	43	9	2	74	

## Data Availability

The data presented in this study are available on request from the corresponding author.
